# Management of Ectopically Erupting Maxillary Incisors: A Case Series

**DOI:** 10.5005/jp-journals-10005-1319

**Published:** 2015-09-11

**Authors:** Kotumachagi Sangappa Suresh, HL Uma, J Nagarathna, Pravin Kumar

**Affiliations:** Professor and Head, Department of Pedodontics and Preventive Dentistry Government Dental College and Research Institute, Bengaluru Karnataka, India; Associate Professor, Department of Orthodontics, Government Dental College and Research Institute, Bengaluru, Karnataka, India; Lecturer, Department of Pedodontics and Preventive Dentistry Government Dental College and Research Institute, Bengaluru Karnataka, India; Postgraduate, Department of Pedodontics and Preventive Dentistry Government Dental College and Research Institute, Bengaluru Karnataka, India

**Keywords:** Ectopic tooth, Eruption disturbances, Fixed orthodontics.

## Abstract

Eruption disturbances related to the position include ectopic eruption and transpositions. The occurrence of ectopic eruption is most commonly associated with maxillary incisors. The normal eruption, position and morphology of these teeth are crucial to craniofacial development, facial esthetics as well as phonetics. It is essential that the clinicians have thorough knowledge of the eruption disturbances in order to make an appropriate, as well as timely intervention, as dictated by the complexity of the problem.

**How to cite this article:** Suresh KS, Uma HL, Nagarathna J, Kumar P. Management of Ectopically Erupting Maxillary Incisors: A Case Series. Int J Clin Pediatr Dent 2015;8(3):227-233.

## INTRODUCTION

Guidance of eruption and development of the primary, mixed and permanent dentitions is an integral component of comprehensive oral healthcare for all pediatric dental patients. Such guidance should contribute to the development of a permanent dentition, i.e. in a stable, functional and esthetically acceptable occlusion.^1^ Incisors are among the first tooth to erupt in both primary and permanent dentition. Maxillary anterior teeth are very important to facial esthetics, often referred to as the ‘social six’, as they are on maximum display during speech and smile in most individuals.

A variety of eruption disturbances arise during the transitional dentition period, which can be broadly classified as: disturbances related to time and disturbances related to position. Ectopic eruption and transposition are disturbances related to position which can cause a delay in eruption time; however, commonly the involved tooth erupts within the expected time frame with an abnormality in position.^2^

Ectopic eruption refers to the eruption of a tooth in a position that is not its normal position in the dental arch.^3^ The prevalence of ectopic eruption is 5.6% and majority of these are permanent central incisors; while maxillary incisors can erupt ectopically or be impacted from supernumerary teeth in up to 2% of the population.^4,5^

Ectopic eruption is considered as a rare developmental anomaly of unknown and controversial etiology. Several theories have been attempted to explain the cause for ectopic eruption. However, the multifactorial process of the growth and development makes it difficult to identify specific primary etiological factors responsible for it.^3^

According to sweet, in 1939, it was related to evolutionary changes which resulted in gradual reduction in the number of permanent teeth of the human dentition. O’Meara stated that insufficient intercanine and anteroposterior growth of the jaws contribute the most.^6^ Niki-foruk and others also share the view of lack of regional bone growth. Several authors have also considered genetic factor to have influence on it.

Following local factors can be probable reasons for ectopic eruption of maxillary incisors:

- Supernumeraries
- Retained deciduous teeth
- Traumatic injury to the primary teeth
- Tooth size arch length discrepancy
- Congenital/developmental disturbance, e.g. cleft of palate, single tooth macrodontia.^7^

Once a tooth (or teeth) is noticed to be ‘ectopically’ erupting, interceptive orthodontics should be carried out in order to reduce the severity of the developing maloc-clusion and the management depends on the etiology, position, patient’s esthetic concern and accommodability of the tooth into an acceptable position within the arch.

Treatment options include:

- Observation for spontaneous correction after removal of the etiological agent.^2^
- Orthodontic intervention by means of either removable or fixed appliance in cases where the ectopically erupted incisors need assistance to be brought into correct position.

Interceptive procedures should be undertaken as soon as sufficient permanent teeth have erupted, as well as co-operation from the child to accept the various steps of the procedure.^5^

The objective of this paper is to report a case series of ectopically erupting maxillary incisors and their management in the mixed dentition.

## CASE REPORTS

### Case 1(A and B)

Children aged 8 (Case 1A) (Fig. 1A) and 7.5 years (Case 1B) (Fig. 2A) were reported to our department with the chief complaint of abnormally erupting tooth in the upper front region. On intraoral examination, it was found that retained deciduous tooth was the etiological agent responsible for ectopic eruption of 21. In both cases, the etiological agent was removed and the patients were kept under observation. Within a period of 3 months, the teeth were aligned in their correct position (Figs 1B and 2B).

**Fig. 1A:**
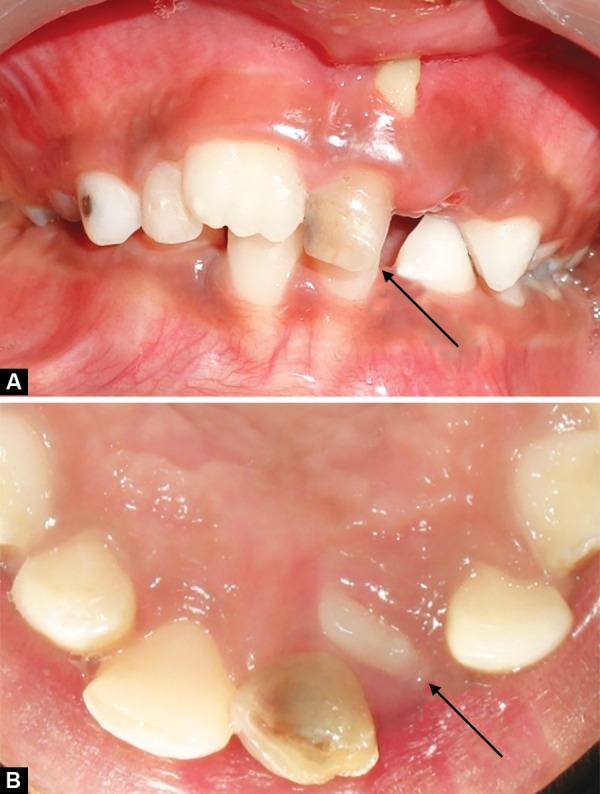
Intraoral frontal and occlusal view showing presence of retained 61 and ectopically erupting 21 (Case 1A)

**Fig. 1B:**
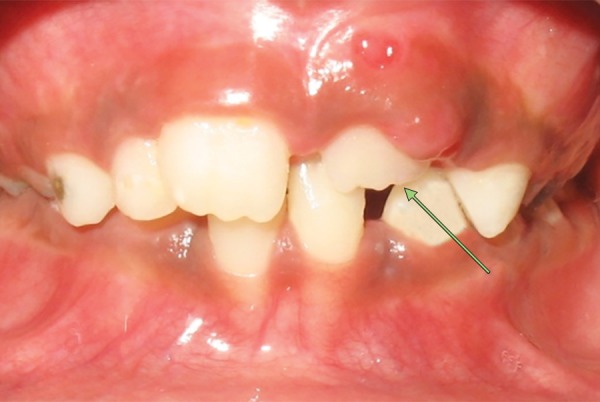
Self-correction of ectopically erupted 21 after extraction of retained 61 (Case 1A)

### Case 2

An 8-year-old boy was reported to the department of pediatric dentistry with the chief complaint of a funny looking small tooth between the upper front teeth region. There was neither a significant medical history nor past dental history.

**Fig. 2A:**
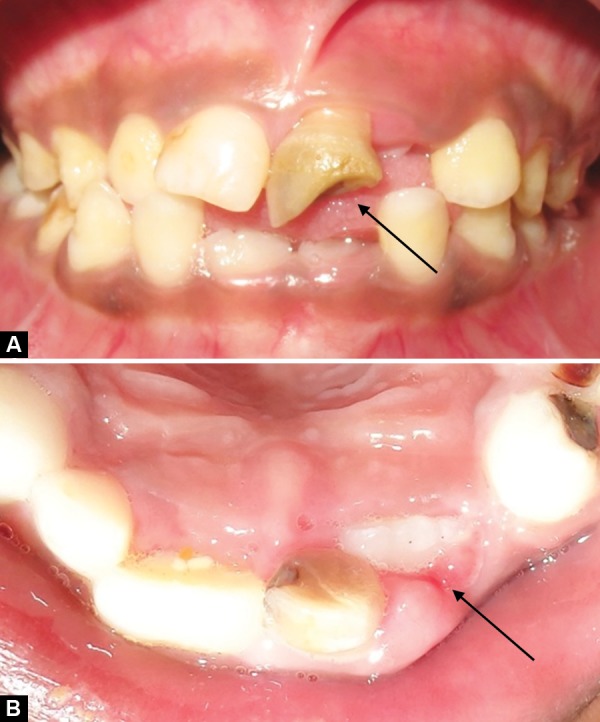
Intraoral frontal and occlusal view showing presence of retained 61 and ectopically erupting 21 (Case 1B)

**Fig. 2B:**
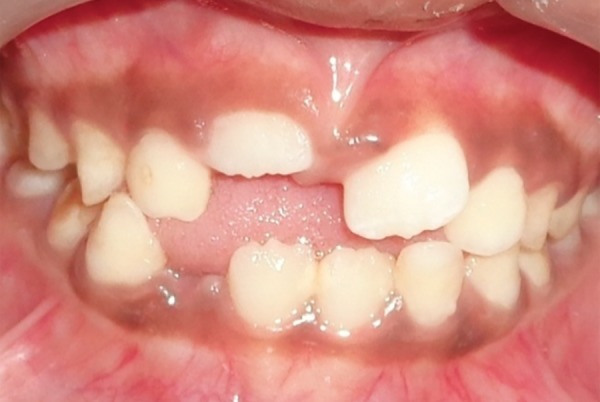
Self-correction of ectopically erupted 21 after extraction of retained 61 (Case 1B)

Intraoral examination revealed early mixed dentition with erupted mesiodens between the upper permanent central incisors. Upper left central incisor was highly placed in the labial sulcus and exhibited distopalatal rotation (Fig. 3A). According to the treatment plan, erupted mesiodens was extracted for fixed orthodontic intervention (Fig. 3B). Both the upper central incisors were banded with welded begg brackets and elastics were placed to close the extracted space (Fig. 3C). Later, a 2 × 2 appliance was planned to align 21 in its correct position, and an uprighting spring was placed on 21 to correct the axial inclination (Fig. 3D). At the next visit 2 weeks later, the tooth was aligned in the correct position (Fig. 3E). The arch wire sequence was 0.016 nickel titanium, 0.016 stainless steel followed by 0.018 stainless steel. After total active treatment time of 4 weeks, the appliance was deboned and a fixed palatal retainer was bonded, which resulted in stable occlusion within 3 months span. The position remained stable after 18 months of the follow-up (Fig. 3F).

**Fig. 3A:**
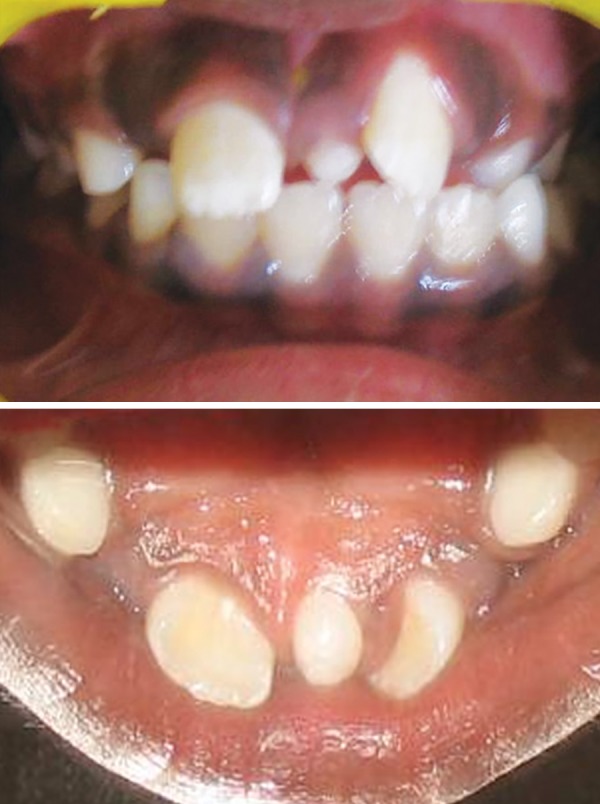
Labial and occlusal view showing ectopically positioned 21 due to presence of the erupted mesiodens (Case 2)

**Fig. 3C:**
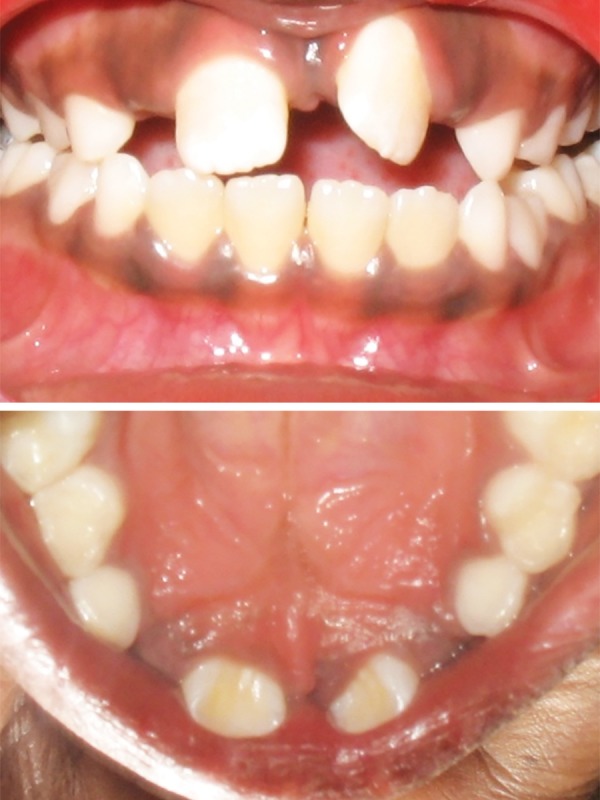
Correction of midline spacing with begg brackets and elastics (Case 2)

### Case 3

A healthy 7-year-old boy was brought by his mother to the department of pediatric dentistry with the complaint of small conical tooth in upper front region. The patient had no significant medical and dental history and intra-oral examination revealed an erupted mesiodens and ectopically erupting 21 (Fig. 4A).

**Fig. 3B:**
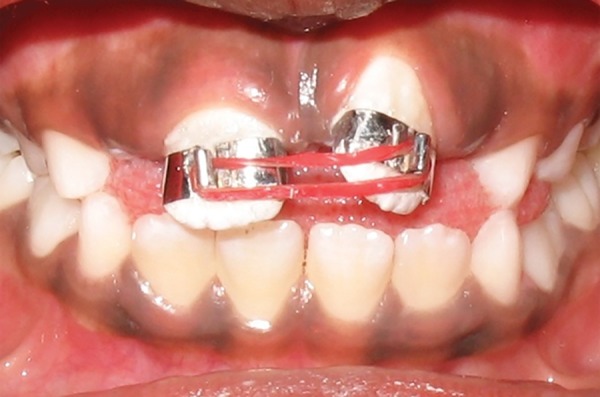
Labial and occlusal view showing persistence of ectopically positioned 21 after 2 months of extraction of mesiodens and large midline spacing (Case 2)

**Fig. 3D:**
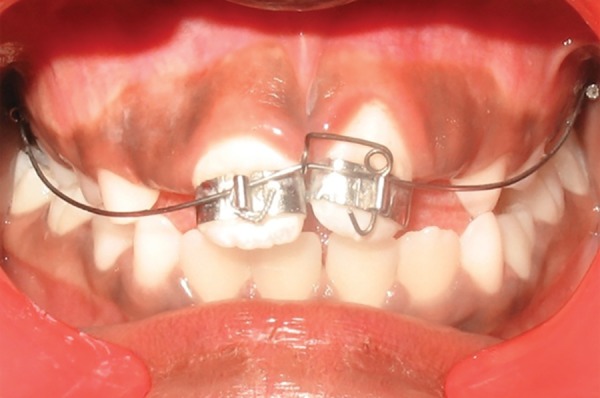
Correction of ectopically positioned 21 with uprighting spring in 2 × 2 appliance (Case 2)

**Fig. 3E:**
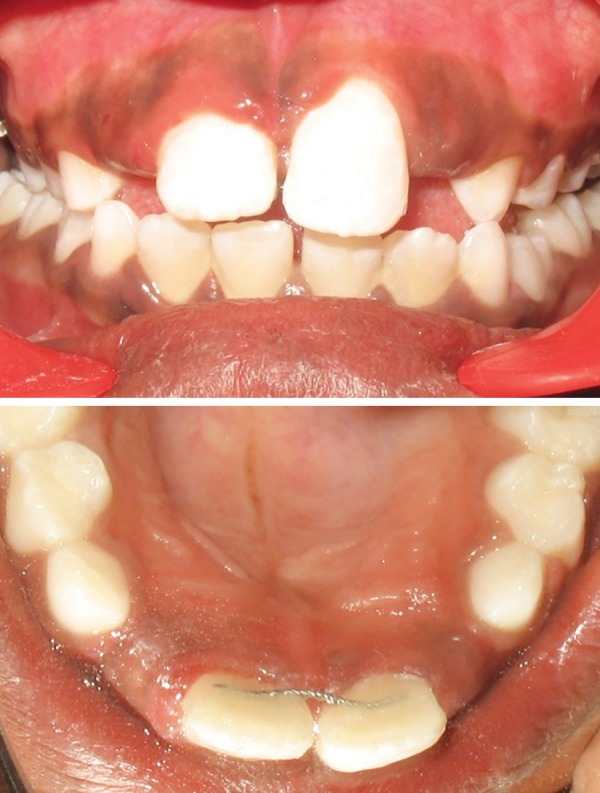
Frontal and occlusal view after alignment (Case 2)

The supernumerary tooth was removed and a course of 2 × 2 appliance therapy was planned. Both the central incisors were bonded with begg brackets and both the upper second deciduous molars were banded and a 0.016 nickel titanium archwire was placed (Fig. 4B). The arch wire sequence followed was: 0.016 nickel titanium, 0.016 and 0.018 stainless steel. The appliance was deboned after alignment of the teeth which took a total duration of 6 weeks. The position remained stable after 18 months of the follow-up.

**Fig. 3F:**
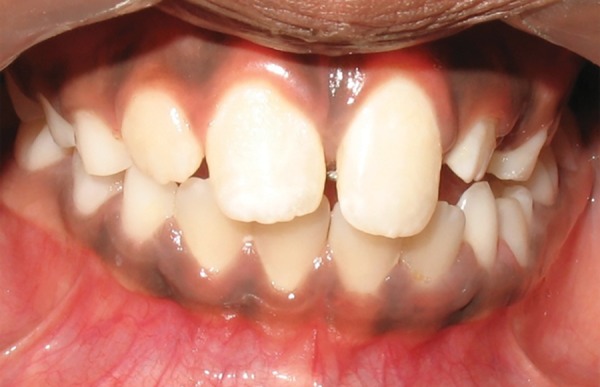
Intraoral view after 18 months post-treatment (Case 2)

**Fig. 4A:**
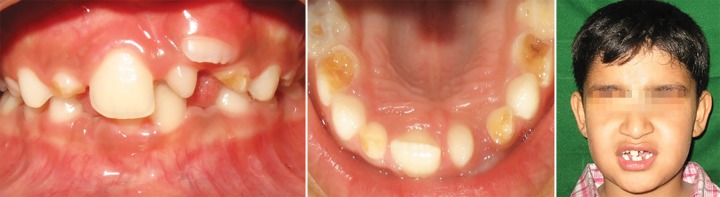
Pretreatment view showing ectopically positioned 21 and erupted mesiodens (Case 3)

**Fig. 4B:**
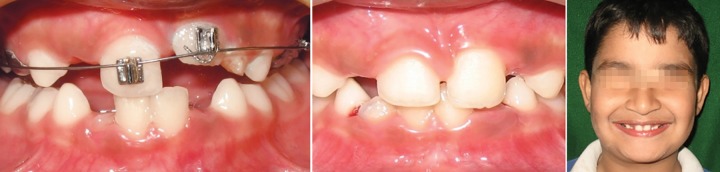
Correction of the ectopically positioned tooth by 2 × 2 Beggs appliance (Case 3)

### Case 4

An 8-year-old girl with her mother presented to our department with the parental concern of abnormally erupting upper front tooth. The past dental history revealed extraction of retained deciduous left upper central incisor 2 weeks ago at a private clinic, and her medical history was unremarkable.

Intraoral examination revealed ectopically, erupting 21, which was erupting high in the labial sulcus (Fig. 5A). Both the upper-central incisors were bonded with begg brackets and both upper first permanent molars were bonded with soldered stainless steel wire, with a hook in 21 region for orthodontic traction (Fig. 5B). Elastics were placed from the hook to the brackets of 21. Later, to align the 21, a 0.016" nickel titanium archwire was placed and left lateral incisor was bracketed after it had erupted sufficiently for additional anchorage. The arch wire sequence followed was: 0.016 nickel titanium, 0.016 and 0.018 stainless steel.

**Fig. 5A:**
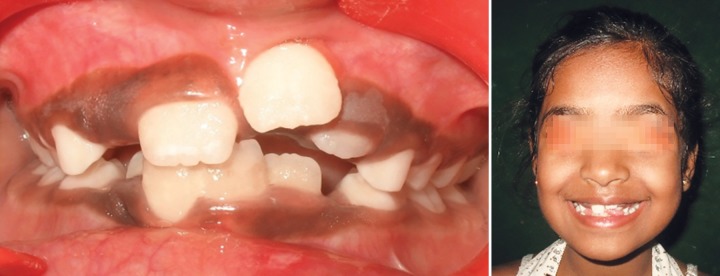
Pretreatment view showing ectopically positioned 21 (Case 4)

**Fig. 5B:**
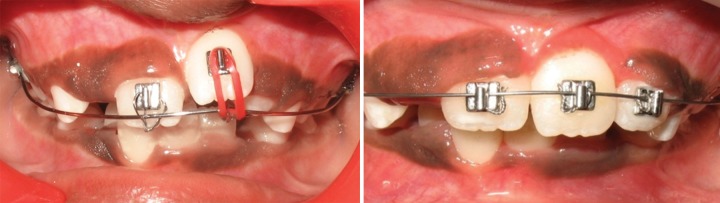
Correction of the ectopically erupted 21 by 2 × 2 fixed appliance (Case 4)

**Fig. 5C:**
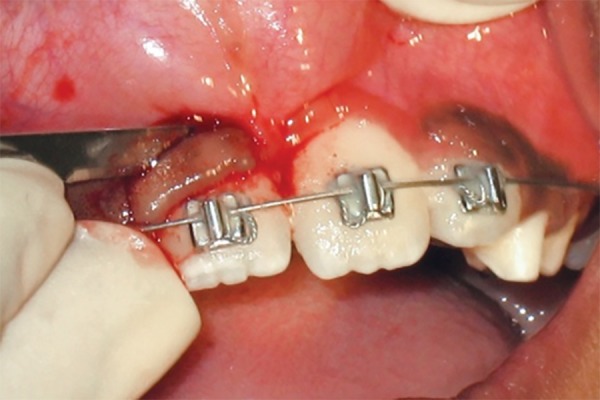
Gingivectomy performed for the correction of gingival contour with respect to 11 (Case 4)

Once the tooth was aligned, gingivectomy was performed with respect to 11, to improve the gingival contour (Fig. 5C). The fixed appliance was debonded after 4 months of active treatment and there was no indication of retainer. The results were stable at 1 year of post-treatment follow-up (Fig. 5D).

**Fig. 5D:**
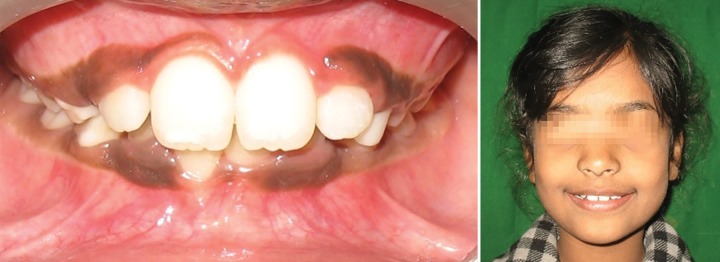
Post-treatment view (Case 4)

## DISCUSSION

It is not uncommon for children to present with variations in normal eruptive patterns of the maxillary incisors. By virtue of the location of the maxillary incisors, parents are often discouraged when eruption patterns do not follow the norms; and will usually prompt the parent to seek treatment to prevent psychological ramifications that accompany abnormalities of the anterior maxilla. To properly treat these individuals, the clinician must have knowledge of the etiology, classification and timely intervention treatment modalities in managing the ectopic eruption of the maxillary incisors.

Dental ectopia is more frequently seen in girls, but according to Huber there is no evidence for sex predic-tion.^8,9^ In this case series also, no female sex prediction for dental ectopia were evident. In the maxilla, occurrence of ectopia is usually unilateral; the present case series also support the same.

Ectopic eruption of permanent incisors can be suspected commonly after trauma to primary incisors, with pulpally-treated primary incisors, retained deciduous teeth, asymmetric eruption and presence of supernumerary tooth.^10-12^

Some ectopically erupted permanent incisors can spontaneously get corrected after removal of the etiologi-cal agent, but others persist or even deteriorate.^13^

In cases 1A and B, retained deciduous tooth was the cause for ectopic eruption of the respective permanent tooth. Due to accelerated stage of eruption and existence of sufficient space in the arch, permanent incisors could align themselves within 3 months without any active treatment, after removal of the etiological agent.

In cases 2 and 3, the presence of an extra tooth was the prime etiological agent for incisor malposition. Supernumerary teeth most commonly disrupt the normal position and eruption of the adjacent teeth, and results in impaction, crowding, diastema, delayed eruption or ectopic eruption; and often require immediate clinical intervention.^14^ The most common location for supernumerary teeth is in the anterior maxilla, which is often observed around 6 to 7 years, either during a routine examination or when disturbances of eruption are noticed. Since the incisors were almost erupted (Case 2) and very labially placed (Case 3), fixed orthodontic intervention was carried out by means of 2 × 2 appliance after removal of the supernumerary teeth.

In case 4, the retained deciduous tooth was the cause of malposition of the upper left central incisor, resulting in eruption of the tooth high in the labial sulcus. Hence, fixed orthodontic intervention by 2 × 2 appliance was carried out. As abnormally positioned incisors are moved into the arch, discrepancies are often observed between the gingival levels of the affected and neighboring teeth. In this case, the gingival contour of the central incisor was brought close to the level of the adjacent central incisor by gingival plastic surgery.

Therefore, evaluating the possibility of self-correction of abnormally erupting incisors (Case 1) at an early stage may enable clinicians to distinguish between the children who require early treatment from those who do not. Each case must be treated independently in order to formulate the proper treatment plan and to achieve the best possible outcome for each patient. Treatment carried out in the early mixed dentition stage with 2 × 2 appliance for cases 2, 3 and 4 had taken a couple of weeks, but the end results were more effectively and efficiently achieved, than if a removable appliance was used.

The 2 × 2 fixed bracketing technique is comprised of brackets on the erupted maxillary incisors, bands on the first permanent maxillary molars and a continuous arch wire. It is used in the early mixed dentition for treatment of both anterior crossbites and alignment of ectopic incisors. This technique offers many advantages as it provides complete control of anterior tooth position, is extremely well tolerated, requires no adjustment by the patient, and allows accurate and rapid positioning of the teeth. The present case series outlined the effectiveness and versatility of the light labial arch technique, and the treatment objectives are achieved within a short period of time. Definitive treatment will probably still be necessary in the permanent dentition, but the complexity and duration of this may be significantly reduced.

In this reported case series, the treatment of ectopically erupting maxillary incisors fulfilled the expected objectives of interceptive treatment by preventing the existing problems from deteriorating, providing a more favorable environment for normal growth and improving facial esthetics for more normal psychosocial development.
